# Direct-Acting Antiviral Therapy and Long-Term Outcomes in Dialysis Patients With Hepatitis C: A Real-world Cohort Study

**DOI:** 10.1093/ofid/ofag389

**Published:** 2026-07-07

**Authors:** Ming-Yan Jiang, Jheng-Yan Wu, Yi-Chan Lee, Tsung-Hsueh Lu

**Affiliations:** Department of Public Health, College of Medicine, National Cheng Kung University, Tainan, Taiwan; Renal Division, Department of Internal Medicine, Chi Mei Medical Center, Tainan, Taiwan; Department of Pharmacy, Chia Nan University of Pharmacy & Science, Tainan, Taiwan; Department of Public Health, College of Medicine, National Cheng Kung University, Tainan, Taiwan; Department of Nutrition, Chi Mei Medical Center, Tainan, Taiwan; Institute of Health Policy and Management, College of Public Health, National Taiwan University, Taipei, Taiwan; Department of Public Health, College of Medicine, National Cheng Kung University, Tainan, Taiwan

**Keywords:** dialysis, direct-acting antivirals, end-stage kidney disease, hepatitis C, mortality

## Abstract

**Background:**

Hepatitis C virus (HCV) infection is associated with adverse outcomes among patients with end-stage kidney disease (ESKD) receiving dialysis. Although direct-acting antivirals (DAAs) achieve high cure rates in patients with ESKD, real-world evidence regarding their long-term clinical benefits remains limited. We evaluated the association between direct-acting antiviral (DAA) therapy and long-term outcomes among dialysis patients with HCV infection.

**Method:**

We conducted a multicenter retrospective cohort study using the TriNetX research network. Adults (≥18 years) with ESKD receiving dialysis and confirmed HCV infection between 2015 and 2025 were included. Patients were classified as DAA-treated or untreated. The primary outcome was all-cause mortality; secondary outcomes included kidney transplantation, incident cirrhosis, and hepatocellular carcinoma. Propensity score matching (1:1) was performed to balance baseline covariates. Outcomes were analyzed using Cox proportional hazards models with up to 5 years of follow-up.

**Results:**

Among 7660 patients (1482 DAA-treated and 6178 untreated), 1458 matched pairs were included in the analysis. DAA therapy was associated with lower mortality (HR, 0.68; 95% CI, 0.59–0.77; *P* < .001) and a higher likelihood of kidney transplantation (hazard ratio [HR], 1.42; 95% confidence interval [CI], 1.12–1.80; *P* = .004). No significant differences were observed in incident cirrhosis or hepatocellular carcinoma during follow-up. Findings were generally consistent across prespecified subgroups.

**Conclusions:**

Among patients with ESKD receiving dialysis and HCV infection, DAA therapy was associated with improved long-term survival and a higher likelihood of kidney transplantation. These findings provide real-world evidence supporting the clinical benefits of antiviral therapy and highlight the importance of expanding HCV treatment in dialysis populations.

Hepatitis C virus (HCV) infection is highly prevalent among individuals with end-stage kidney disease (ESKD), affecting approximately 20% of patients receiving hemodialysis worldwide [1–3]. Chronic HCV infection in this population is associated with reduced quality of life [4], increased morbidity and mortality [5], and inferior outcomes after kidney transplantation, including higher patient mortality and poorer graft survival [6–10]. Historically, HCV infection has posed a major barrier to kidney transplantation, with many patients experiencing delayed wait-listing or exclusion from transplant eligibility due to concerns about posttransplant outcomes [11].

The advent of direct-acting antivirals (DAAs) has revolutionized HCV treatment, offering high cure rates, short treatment durations, and excellent tolerability compared with interferon-based therapies [12]. In the general population, viral eradication with DAAs has been associated with improved survival and reduced hepatic and extrahepatic complications [13, 14]. However, the long-term clinical benefits of antiviral therapy in patients receiving dialysis remain uncertain [15]. Evidence from the interferon era suggested improved survival with antiviral therapy in hemodialysis populations [16, 17]. More recent studies of direct-acting antiviral (DAA) therapy in patients with end-stage kidney disease (ESKD) have largely focused on sustained virologic response (SVR) or short-term surrogate outcomes, with few investigations evaluating long-term clinical endpoints such as mortality or kidney transplantation [18]. In addition, existing studies are often limited by small sample sizes and relatively short follow-up periods [18].

Despite the demonstrated high efficacy of DAAs, contemporary evidence on their long-term benefits in dialysis patients remains limited, with much of the existing literature derived from the interferon era [19]. Given the substantial burden of HCV infection in ESKD, the expanding use of DAAs, and the unique risk profile of dialysis patients, robust data are needed to clarify the long-term clinical benefits of antiviral therapy. Accordingly, we used the TriNetX global collaborative research network, a large real-world database, to evaluate the long-term association between DAA therapy and clinical outcomes, including survival and kidney transplantation, among patients with ESKD and HCV infection.

## METHODS

### Data Source and Study Population

We conducted a multicenter retrospective cohort study using the TriNetX research network (TriNetX, LLC), a federated global health platform that aggregates de-identified electronic health records and insurance claims from over 140 healthcare organizations across multiple countries, encompassing approximately 150 million patients [20]. Diagnoses, procedures, and laboratory data were identified using International Classification of Diseases (ICD), Current Procedural Terminology (CPT), and Logical Observation Identifiers Names and Codes (LOINC) codes, respectively. Medication data were derived from prescription and medication records and categorized using the Veterans Affairs Drug Classification System. TriNetX data are de-identified in accordance with the HIPAA Privacy Rule using expert determination.

Adult patients (≥18 years) with confirmed HCV infection, defined by a positive HCV RNA test, and ESKD receiving dialysis, defined by ICD-10 codes N18.6 or Z99.2, were identified in the TriNetX network (Supplementary Table 1). The study included patients who either initiated DAA therapy or had no documented DAA prescriptions during the study period (January 2015–May 2025). Patients were categorized into DAA-treated and untreated cohorts using an intention-to-treat framework. The treated cohort included patients receiving at least 1 DAA prescription after confirmation of HCV viremia, regardless of treatment completion or documented viral clearance. DAA regimens included commonly used agents, such as daclatasvir, asunaprevir, ombitasvir, paritaprevir, ritonavir, dasabuvir, elbasvir, grazoprevir, ledipasvir, sofosbuvir, glecaprevir, pibrentasvir, velpatasvir, or voxilaprevir, and related combinations. The untreated cohort consisted of patients without documented DAA prescriptions during the follow-up period. Patients with kidney transplantation prior to cohort entry were excluded. Data extraction was completed in August 2025.

### Patient Consent Statement

Patient consent was waived because this study used de-identified, secondary data from the TriNetX research network. No individual patient identifiers were accessed, and all analyses were conducted in accordance with relevant guidelines and regulations.

### Covariates

Baseline variables included demographics (age, sex, race/ethnicity), lifestyle factors (smoking, alcohol use), overweight or obesity, and comorbidities including hypertension, dyslipidemia, ischemic heart disease, heart failure, diabetes with complications, neoplasm, hepatocellular carcinoma, cirrhosis, prior liver transplant, human immunodeficiency virus infection, and chronic hepatitis B (Supplementary Table 2). Laboratory parameters included anemia (hemoglobin <10 g/dL), hypoalbuminemia (albumin <3.5 g/dL), thrombocytopenia (platelet count <100 × 10^3^/µL), elevated liver enzymes (AST or ALT ≥50 IU/L), and poor glycemic control (HbA1c ≥ 8%) (Supplementary Table 2).

### Outcomes

The primary outcome was all-cause mortality. Secondary outcomes included kidney transplantation, new-onset cirrhosis, and incident hepatocellular carcinoma, identified using diagnostic and procedural codes (Supplementary Table 3). For the analyses of incident cirrhosis and hepatocellular carcinoma, patients with a documented diagnosis of these conditions prior to or on the index date were excluded to ensure the evaluation of new-onset disease. Exploratory outcomes included changes in serum albumin and hemoglobin levels at 12 months post-index. Follow-up began the day after the index date and continued until the outcome event, 60 months after the index date, or 31 May 2025, whichever occurred first. For treated patients, the index date was defined as the date of the first DAA prescription, whereas for untreated patients it was defined as the first recorded positive HCV RNA test within the study period. Accordingly, cohort entry occurred at varying time points during the study period depending on when these events occurred. This exposure-based definition was used to align cohort entry and follow-up time with treatment initiation while minimizing immortal time bias and avoiding misclassification of untreated person-time as exposed time.

### Sensitivity and Subgroup Analyses

To mitigate immortal time bias, prespecified landmark analyses were conducted at 6 and 12 months after the index date. Follow-up time was reinitiated at the landmark time point, and outcomes were evaluated conditionally among patients remaining alive at that time. For each landmark analysis (6-month and 12-month), propensity score matching (PSM) was reperformed among patients who remained alive at the respective landmark time point.

A per-protocol-like sensitivity analysis was performed among patients with available follow-up HCV RNA testing to evaluate the association between documented viral clearance and survival. Patients with persistent viremia were defined as those with either a second positive HCV RNA test within the 6–12-month window after the initial positive test or at least 2 positive HCV RNA tests ≥6 months apart in the absence of DAA exposure. This group was compared with DAA-treated patients who had a documented negative HCV RNA test within the 6–12-month window or ≥6 months after treatment initiation (SVR proxy), using the same time windows for follow-up RNA assessment.

Prespecified subgroup analyses were stratified by sex, age (<65 vs ≥65 years), baseline liver disease status (presence vs absence of cirrhosis or hepatocellular carcinoma), and HBV and HIV coinfection statuses.

### Statistical Analysis

Baseline characteristics were summarized as means with standard deviations for continuous variables and counts with percentages for categorical variables. Differences between groups were assessed using *t*-tests or chi-square tests.

To reduce confounding, 1:1 propensity score matching was performed using all prespecified covariates. Propensity scores were estimated using logistic regression and matched with a greedy nearest-neighbor algorithm with a caliper width of 0.1 pooled standard deviations. Covariate balance was assessed using standardized mean differences (SMD), with values <0.1 indicating acceptable balance.

Time-to-event outcomes were evaluated using Kaplan-Meier survival curves with log-rank tests. Hazard ratios (HRs) and 95% confidence intervals (CIs) were estimated using Cox proportional hazards models. Cause-specific Cox proportional hazards models were performed for nonfatal outcomes, as the TriNetX platform does not support formal competing risk regression methods (eg, Fine-Gray subdistribution hazard models). Nested multivariable Cox models were used to progressively adjust for demographic, clinical, laboratory, and diagnostic covariates. Two-sided *P* values <.05 were considered statistically significant.

Baseline albumin and hemoglobin were incorporated into the propensity score matching algorithm as categorical variables using clinically relevant thresholds (3.5 and 10 g/dL, respectively) in accordance with the limitations of the TriNetX platform, which does not permit matching on continuous variables. The 12-month post-index changes in these markers were analyzed and compared as continuous variables.

All analyses were performed using validated statistical tools within the TriNetX analytics platform [20, 21].

## RESULTS

### Study Cohort

A total of 7660 patients met the inclusion criteria, including 1482 DAA-treated and 6178 untreated patients, representing a large multicenter global cohort of dialysis patients with HCV infection. The study cohort was predominantly derived from the United States, with smaller contributions from the Asia-Pacific and Europe/Middle East/Africa regions (Supplementary Table 4). Analysis of treatment regimens showed patterns consistent with contemporary interferon-free HCV treatment practices (Supplementary Table 5). The most frequently prescribed agents were sofosbuvir (39.2%), glecaprevir/pibrentasvir (33.8%), and velpatasvir (29.2%). Pangenotypic interferon-free regimens, including glecaprevir/pibrentasvir and sofosbuvir/velpatasvir-based therapies, accounted for approximately 63.4% of treatments, genotype-specific interferon-free regimens accounted for approximately 33.2%, and early-generation DAA regimens for fewer than 3.5%.

Before propensity score matching (PSM), DAA-treated patients were slightly older than untreated patients (mean [SD] age, 59.0 [10.3] vs 57.3 [12.5] years; *P* < .001). Approximately two-thirds of the cohort were male and about half were African American. DAA-treated patients had higher prevalences of several comorbidities, including diabetes, hypertension, cardiovascular disease, cirrhosis, hepatocellular carcinoma, chronic hepatitis B, and HIV infection, but generally showed more favorable laboratory profiles, including higher albumin and hemoglobin levels. After 1:1 PSM, 2916 patients (1458 matched pairs) were included in the final analysis, and all matching covariates achieved acceptable balance using prespecified categorical definitions (SMD <0.1) (Table 1).

**Table 1.** Baseline Characteristics of Patients Treated With Direct-Acting Antivirals (DAAs) [DAA (+)] and Untreated Patients [DAA (−)] Before and After Propensity Score Matching

	Before Matching			After Matching		
	DAA (+)	DAA (−)	SMD	DAA (+)	DAA (−)	SMD
	(n = 1482)	(n = 6178)		(n = 1458)	(n = 1458)	
Demographic data						
Age at index date	59.0 ± 10.3	57.3 ± 12.5	0.147	59.0 ± 10.3	58.9 ± 12.0	0.010
Male	993 (67.0%)	4053 (65.6%)	0.030	975 (66.9%)	992 (68.0%)	0.025
Race	…	…	…	…	…	…
Black/African American	676 (45.6%)	3174 (51.4%)	0.115	668 (45.8%)	652 (44.7%)	0.022
White	501 (33.8%)	1980 (32.0%)	0.037	491 (33.7%)	502 (34.4%)	0.016
Asian	128 (8.6%)	223 (3.6%)	0.211	126 (8.6%)	127 (8.7%)	0.002
Other race	47 (3.2%)	328 (5.3%)	0.106	46 (3.2%)	52 (3.6%)	0.023
Unknown race	113 (7.6%)	427 (6.9%)	0.027	110 (7.5%)	112 (7.7%)	0.005
Overweight/obesity	352 (23.8%)	1213 (19.6%)	0.100	349 (23.9%)	356 (24.4%)	0.011
Smoking	564 (38.1%)	1832 (29.7%)	0.178	554 (38.0%)	554 (38.0%)	<0.001
Alcohol use	336 (22.7%)	844 (13.7%)	0.235	328 (22.5%)	328 (22.5%)	<0.001
Comorbidity						
Type 2 diabetes mellitus	805 (54.3%)	2789 (45.1%)	0.184	789 (54.1%)	811 (55.6%)	0.030
Diabetic kidney complication	618 (41.7%)	2163 (35.0%)	0.138	608 (41.7%)	626 (42.9%)	0.025
Diabetic ophthalmic complication	194 (13.1%)	582 (9.4%)	0.116	190 (13.0%)	197 (13.5%)	0.014
Diabetic neurological complication	274 (18.5%)	925 (15.0%)	0.094	270 (18.5%)	286 (19.6%)	0.028
Diabetic circulatory complication	107 (7.2%)	451 (7.3%)	0.003	107 (7.3%)	104 (7.1%)	0.008
Hypertension	1154 (77.9%)	3539 (54.3%)	0.451	1132 (77.6%)	1158 (79.4%)	0.043
Dyslipidemia	697 (47.0%)	2215 (35.9%)	0.228	687 (47.1%)	686 (47.1%)	0.001
Heart failure	613 (41.4%)	2087 (33.8%)	0.157	606 (41.6%)	637 (43.7%)	0.043
Ischemic heart disease	631 (42.6%)	2244 (36.3%)	0.128	625 (42.9%)	655 (44.9%)	0.041
Neoplasm	627 (42.3%)	1401 (22.7%)	0.429	610 (41.8%)	589 (40.4%)	0.029
Hepatocellular carcinoma	120 (8.1%)	196 (3.2%)	0.215	112 (7.7%)	107 (7.3%)	0.013
Cirrhosis	571 (38.5%)	1125 (18.2%)	0.463	551 (37.8%)	522 (35.8%)	0.041
Liver transplantation	160 (10.8%)	179 (2.9%)	0.317	152 (10.4%)	127 (8.7%)	0.058
Human immunodeficiency virus	152 (10.3%)	184 (3.0%)	0.296	139 (9.5%)	132 (9.1%)	0.017
Hepatitis B virus	78 (5.3%)	111 (1.8%)	0.189	71 (4.9%)	73 (5.0%)	0.006
Laboratory data of blood						
Hemoglobin <10 g/dL	1046 (70.6%)	3385 (54.8%)	0.331	1026 (70.4%)	1016 (69.7%)	0.015
Hemoglobin levels (g/dL)	10.9 ± 2.2	10.1 ± 2.3	0.390	10.9 ± 2.2	10.3 ± 2.3	0.292
Albumin <3.5 g/dL	1118 (75.4%)	3594 (58.2%)	0.373	1098 (75.3%)	1114 (76.4%)	0.026
Albumin levels (g/dL)	3.5 ± 0.7	3.2 ± 0.8	0.404	3.5 ± 0.7	3.3 ± 0.8	0.344
Platelet ≥100 × 10^3^/µL	1388 (93.7%)	4342 (70.3%)	0.638	1364 (93.6%)	1394 (95.6%)	0.091
Platelet counts (10^3^/µL)	181.9 ± 86.8	187.8 ± 102.4	0.062	182.3 ± 86.9	179.0 ± 92.1	0.037
Aspartate aminotransferase ≥50 U/L	874 (59.0%)	2383 (38.6%)	0.417	854 (58.6%)	858 (58.8%)	0.006
Aspartate aminotransferase levels (U/L)	39.8 ± 39.7	89.2 ± 431.3	0.161	39.4 ± 38.4	76.9 ± 208.2	0.251
Alanine transaminase ≥50 U/L	828 (55.9%)	1949 (31.5%)	0.506	806 (55.3%)	829 (56.9%)	0.032
Alanine transaminase levels (U/L)	37.4 ± 49.2	61.0 ± 243.9	0.134	36.7 ± 44.6	56.2 ± 131.1	0.199
Hemoglobin A1c<8%	1003 (67.7%)	2565 (41.5%)	0.545	980 (67.2%)	1001 (68.7%)	0.031
Hemoglobin A1c levels (%)	6.0 ± 1.7	6.3 ± 1.9	0.131	6.0 ± 1.7	6.0 ± 1.7	<0.001

Abbreviation: SMD, standardized mean difference.

### Primary Outcome

**Figure 1.**
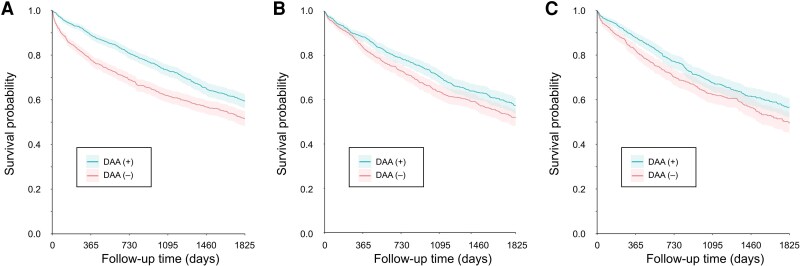
Kaplan-Meier survival curves for all-cause mortality in dialysis patients with hepatitis C virus (HCV) infection, comparing those treated with direct-acting antivirals (DAAs) versus untreated. *A*, Analysis of the overall cohort from the index date. *B*, Six-month landmark analysis including only patients who survived ≥6 months after the index date. Survival follow-up was reinitiated at the 6-month landmark time point, and curves represent conditional survival thereafter. *C*, Twelve-month landmark analysis including only patients who survived ≥12 months after the index date. Survival follow-up was reinitiated at the 12-month landmark time point, and curves represent conditional survival thereafter. In all 3 analyses, DAA therapy was associated with a significantly higher cumulative survival probability (*P* < .01).

In the matched cohort, the median follow-up duration was 1056 days in the DAA-treated group and 645 days in the untreated group. During a follow-up of up to 5 years, 429 deaths (29.4%) occurred among DAA-treated patients compared with 505 deaths (34.6%) among untreated patients, yielding an absolute risk reduction of 5.2% (95% CI, −8.6% to −1.8%; *P* < .01). Using a nested modeling approach in model 1 (adjusted only for age, sex, and race), DAA therapy was associated with a 32% lower hazard of mortality (HR, 0.68; 95% CI, 0.60–0.77; *P* < .001) (Table 2). The results remained consistent across model 2 through Model 4, which progressively incorporated baseline comorbidities and laboratory markers. In the fully adjusted model (model 5), which accounted for the complete set of demographic, clinical, and laboratory variables, DAA therapy continued to demonstrate a significant 32% lower hazard of all-cause mortality (HR, 0.68; 95% CI, 0.59–0.77; *P* < .001) (Table 2 and Figure 1*A*).

**Table 2.** Hazard Ratios for All-cause Mortality and Kidney Transplantation Over 5-Year Follow-up Between Patients Treated With Direct-Acting Antivirals [DAA (+)] and Untreated Patients [DAA (−)]

	All-Cause Mortality HR (95% CI)	*P* Value	Kidney Transplantation HR (95% CI)	*P* Value
Model 1	0.68 (0.60–0.77)	<.001	0.96 (0.78–1.20)	.74
Model 2	0.59 (0.52–0.66)	<.001	1.04 (0.83–1.29)	.74
Model 3	0.58 (0.51–0.65)	<.001	1.38 (1.09–1.75)	.007
Model 4	0.63 (0.56–0.71)	<.001	1.28 (1.01–1.61)	.04
Model 5	0.68 (0.59–0.77)	<.001	1.42 (1.12–1.80)	.003

Model descriptions:

Model 1: adjusted for demographic variables (age, sex, and race).

Model 2: adjusted for model 1 variables plus primary comorbidities (diabetes, hypertension, cirrhosis, and hepatocellular carcinoma).

Model 3: adjusted for model 2 variables plus additional lifestyle and clinical factors (dyslipidemia, heart failure, ischemic heart disease, neoplasm, smoking, alcohol use, overweight/obesity, liver transplant status, HIV infection, and chronic hepatitis B).

Model 4: adjusted for model 3 variables plus baseline laboratory parameters (hemoglobin, albumin, platelet count, aspartate aminotransferase, alanine aminotransferase, and hemoglobin A1c).

Model 5 (fully adjusted): adjusted for model 4 variables plus specific diabetic complications (renal, ophthalmic, neurological, and circulatory complications).

Abbreviations: CI, confidence interval; HR, hazard ratio.

In the landmark analyses, baseline characteristics remained well balanced after re-matching in both landmark cohorts (Supplementary Tables 6 and 7). Landmark analyses at 6 months (HR, 0.81; 95% CI, 0.70–0.94; *P* < .01) and 12 months (HR, 0.78; 95% CI, 0.66–0.93; *P* < .01) demonstrated that the survival benefit persisted even after excluding early events (Supplementary Table 8; Figure 1*B* and 1*C*).

Sensitivity analyses restricted to patients with available follow-up HCV RNA data yielded consistent results (Supplementary Table 9). Compared with patients with persistent viremia, defined as either a second positive HCV RNA test within the 6–12-month window or ≥2 positive tests ≥6 months apart in the absence of DAA exposure, DAA-treated patients with a documented negative HCV RNA result (SVR proxy) had significantly lower mortality risk when the second RNA measurement occurred within the 6–12-month window (HR, 0.57; 95% CI, 0.38–0.85; *P* < .01) and when it occurred ≥6 months during follow-up (HR, 0.52; 95% CI, 0.37–0.73; *P* < .001) (Supplementary Table 9).

### Secondary Outcomes

Kidney transplantation occurred in 183 of DAA-treated and 111 of untreated patients, an absolute difference of 4.9% (95% CI, 2.8%–7.1%; *P* < .001). In the fully adjusted model, DAA therapy was associated with a significantly higher likelihood of transplantation (HR, 1.42; 95% CI, 1.12–1.80; *P* < .01) (Table 2).

Among patients without baseline cirrhosis or hepatocellular carcinoma, incident cirrhosis or hepatocellular carcinoma occurred in 118 DAA-treated and 100 untreated patients, with no significant difference between groups (Table 3).

**Table 3.** Liver-Related Outcomes and Composite Mortality in Patients Without Pre-existing Advanced Liver Disease (n = 812)

Outcome	HR (95% CI)	*P* Value
Liver-specific events		
Hepatocellular carcinoma	1.32 (0.77–2.25)	.31
Cirrhosis	1.02 (0.72–1.43)	.93
Composite: hepatocellular carcinoma or cirrhosis	1.05 (0.76–1.46)	.77
Composite outcomes including mortality		
Hepatocellular carcinoma or all-cause death	0.70 (0.58–0.85)	<.001
Cirrhosis or all-cause death	0.74 (0.62–0.88)	<.001
Hepatocellular carcinoma, cirrhosis, or all-cause death	0.75 (0.63–0.89)	.001

Analysis restricted to patients without evidence of cirrhosis or hepatocellular carcinoma at baseline (n = 812 per group after matching).

All models are fully adjusted for demographic variables, comorbidities, and baseline laboratory parameters (Model 5 parameters).

The inclusion of all-cause death in composite outcomes accounts for the significant competing risk of mortality in the end-stage kidney disease (ESKD) population, demonstrating the overall clinical benefit of direct-acting antiviral (DAA) therapy even when isolated liver events do not reach statistical significance within the 5-year follow-up.

Abbreviations: CI, confidence interval; HR, hazard ratio.

Exploratory analyses showed that DAA-treated patients had greater improvements in serum albumin and hemoglobin levels at 12 months compared with untreated patients (Supplementary Table 10).

### Subgroup Analysis

The association between DAA therapy and improved survival was consistent across prespecified subgroups (Table 4). Both males and females treated with DAAs had lower risks of all-cause mortality (males, HR 0.68; 95% CI, 0.58–0.80; *P* < .001; females, HR 0.59; 95% CI, 0.47–0.74; *P* < .001) and higher likelihood of kidney transplantation (males, HR 1.50; 95% CI, 1.11–2.01; *P* < .01; females, HR 1.65; 95% CI, 1.05–2.60; *P* < .05).

**Table 4.** Stratified Analysis of All-cause Mortality and Kidney Transplantation Within 5 Years in Patients Treated With Direct-Acting Antivirals Versus Untreated Patients

	All-Cause Mortality HR (95% CI)	*P* Value	Kidney Transplantation HR (95% CI)	*P* Value
Sex				
Male	0.68 (0.58–0.80)	<.001	1.50 (1.11–2.01)	.008
Female	0.59 (0.47–0.74)	<.001	1.65 (1.05–2.60)	.03
Age		<.001		
<65 y old	0.64 (0.51–0.81)	<.001	1.45 (1.02–2.07)	.04
≥65 y old	0.69 (0.59–0.81)	<.001	1.45 (1.05–2.01)	.03
Liver disease status		<.001		
Without cirrhosis/hepatocellular carcinoma	0.70 (0.58–0.84)	<.001	1.88 (1.37–2.57)	<.001
With cirrhosis/hepatocellular carcinoma	0.63 (0.52–0.76)	<.001	1.07 (0.71–1.59)	.76

Incident cirrhosis and hepatocellular carcinoma were not included in the stratified analyses, as no significant differences were observed in the overall cohort (see Table 3).

Abbreviations: CI, confidence interval; HR, hazard ratio.

Similar findings were observed across age groups. Patients younger than 65 years who received DAAs had reduced mortality (HR, 0.64; 95% CI, 0.51–0.81; *P* < .001) and higher transplantation probability (HR, 1.45; 95% CI, 1.02–2.07; *P* < .05), while patients aged ≥65 years also showed lower mortality (HR, 0.69; 95% CI, 0.59–0.81; *P* < .001) and increased transplantation likelihood (HR, 1.45; 95% CI, 1.05–2.01; *P* < .05).

When stratified by liver disease status, DAA therapy was associated with lower mortality in both patients without cirrhosis or hepatocellular carcinoma (HR, 0.70; 95% CI, 0.58–0.84; *P* < .001) and those with these conditions (HR, 0.63; 95% CI, 0.52–0.76; *P* < .001). The increase in transplantation probability was significant only among patients without advanced liver disease. No subgroup showed significant differences in incident cirrhosis or hepatocellular carcinoma.

Additional subgroup analyses stratified by HBV and HIV coinfection status showed generally similar directional associations between DAA therapy and lower mortality risk; however, statistical significance was not consistently observed among coinfected subgroups, likely reflecting limited statistical power due to small sample sizes (Supplementary Table 11).

## DISCUSSION

In this large multicenter real-world cohort of dialysis patients with HCV infection, DAA therapy was associated with a 32% lower risk of long-term all-cause mortality and a 42% higher likelihood of kidney transplantation. Treated patients also showed improvements in albumin and hemoglobin levels, suggesting potential benefits in nutritional and hematologic status. These findings provide real-world evidence supporting the clinical benefits of DAA therapy in patients with ESKD and are particularly relevant to global HCV elimination efforts targeting high-risk populations.

Although DAAs achieve high SVR rates in patients with ESKD [22–24], evidence regarding their long-term survival benefits remains limited. Historically, the slow progression of HCV infection and limited life expectancy in dialysis patients led to uncertainty regarding the net clinical benefit of antiviral therapy, and treatment decisions were often individualized based on transplant candidacy, comorbidity burden, and expected survival [15]. While interferon-era studies suggested improved survival in hemodialysis populations [16, 17], more recent investigations in the DAA era have focused on SVR rates and short-term outcomes rather than long-term clinical endpoints [25–27]. Our study helps fill this evidence gap by demonstrating significant associations with improved long-term survival and a higher likelihood of kidney transplantation, highlighting the broad clinical value of viral eradication in this high-risk population.

Several mechanisms may underlie the association between DAA therapy and improved outcomes. Chronic HCV infection promotes systemic inflammation, immune dysregulation, insulin resistance, and accelerated atherosclerosis, all of which contribute to cardiovascular morbidity and mortality in patients with ESKD [28–30]. Prior studies in dialysis populations suggest that cardiovascular disease and infection are the leading causes of death, although cause-specific mortality data were not reliably available in the TriNetX database. Viral eradication with DAAs may mitigate these processes, thereby improving cardiovascular and metabolic health and ultimately survival [31]. The observed increases in albumin and hemoglobin may further reflect reduced inflammation and improved hepatic synthetic function [32], which are associated with better prognosis in dialysis populations.

Notably, no significant differences were observed in the incidence of cirrhosis or hepatocellular carcinoma. This may reflect the substantial competing risk of mortality in the ESKD population and the relatively limited follow-up duration, which may be insufficient to capture long-term liver-related outcomes. In addition, ascertainment of liver-related outcomes in administrative and electronic health record databases may be incomplete, potentially contributing to outcome misclassification. Therefore, these findings should be interpreted in the context of these methodological limitations and do not necessarily contradict the established hepatic benefits of DAA therapy reported in non-ESKD populations.

Historically, HCV infection represented a major barrier to transplant eligibility because of concerns regarding posttransplant complications and inferior outcomes [11, 33]. In our study, DAA therapy was associated with a higher likelihood of kidney transplantation. Viral eradication may improve hepatic function and reduce systemic inflammation, potentially increasing transplant eligibility. However, patients receiving DAA therapy may have differed in unmeasured characteristics such as healthcare engagement, adherence, and socioeconomic status, and transplant evaluation may also have influenced treatment allocation. Therefore, this finding should be interpreted as an association and does not necessarily imply a causal effect of DAA therapy on transplant access.

Taken together, our findings suggest that DAA therapy provides important clinical benefits for patients with HCV and ESKD beyond viral eradication. These results support routine HCV screening and timely initiation of antiviral therapy in accordance with current guideline recommendations [12]. The widespread availability of DAAs may therefore help improve outcomes and expand access to kidney transplantation in this high-risk population.

A major strength of this study is the use of the global TriNetX research network, which enabled analysis of a large multicenter cohort of 7660 patients across more than 140 healthcare organizations. Propensity score matching was applied to reduce baseline imbalances between treatment groups. In addition, up to 5 years of follow-up allowed evaluation of clinically relevant outcomes, including all-cause mortality and kidney transplantation, in a high-risk population often underrepresented in clinical trials.

However, several limitations should be acknowledged. First, the observational design precludes causal inference, and residual confounding from unmeasured variables, such as frailty, socioeconomic status, health-seeking behavior, or clinician prescribing bias, cannot be excluded. Although propensity score matching achieved good balance for categorical variables, residual imbalance remained for continuous measures such as serum albumin and hemoglobin, reflecting limitations of the TriNetX platform, which does not support matching on continuous distributions.

Second, detailed clinical information, including HCV genotype, fibrosis stage, SVR status, and treatment adherence, was unavailable. Similarly, validated liver disease severity scores such as Child-Pugh classification and model for end-stage liver disease (MELD) score could not be calculated due to the inability to reconstruct individual-level clinical variables within the de-identified federated database. Instead, we used available laboratory surrogates, including serum albumin and platelet count (thrombocytopenia), as indirect markers of hepatic dysfunction and portal hypertension, although these may be imperfect substitutes for validated liver severity scores. In addition, medication exposure was based on prescription records and may not fully reflect actual treatment initiation or completion. Although newer-generation DAAs achieve SVR rates exceeding 95% even in patients with ESKD [22, 34], the absence of direct virologic data limited our ability to directly link viral clearance with clinical outcomes. Our sensitivity analysis using an SVR proxy demonstrated consistent associations with mortality outcomes, supporting the robustness of our primary analysis based on treatment exposure.

Third, although we observed improvements in albumin and hemoglobin levels among DAA-treated patients, these findings remain exploratory, as these markers are influenced by multiple factors in dialysis populations. Fourth, as with all studies using electronic health record data, misclassification and incomplete ascertainment are possible, particularly for liver-related outcomes identified through diagnostic coding across multiple healthcare systems.

Fifth, differences in timing and duration of HCV infection could not be fully accounted for, as the exact date of infection is rarely known. Therefore, index dates were defined based on first HCV RNA positivity (untreated) and DAA initiation (treated) to minimize immortal time bias, although residual heterogeneity in disease duration may remain.

Sixth, competing risk analyses could not be performed due to TriNetX platform limitations; therefore, estimates for nonfatal outcomes should be interpreted cautiously in this high-mortality population. Finally, although our sample was large and multicenter across varying geographic regions, most data were derived from healthcare systems in the United States, which may limit generalizability to regions with different practice patterns or healthcare resources.

In summary, DAA therapy in HCV-infected patients undergoing dialysis was associated with improved long-term survival and increased likelihood of kidney transplantation. These results support current guidelines recommending HCV treatment in individuals with advanced kidney disease and highlight the broader clinical advantages of DAAs beyond viral eradication. Future prospective studies with detailed virologic and clinical data are needed to confirm these associations and further define the role of antiviral therapy in improving outcomes and advancing HCV micro-elimination in the ESKD population.

## Supplementary Material

ofag389_Supplementary_Data

## Supplementary Data

Supplementary materials are available at *Open Forum Infectious Diseases* online. Consisting of data provided by the authors to benefit the reader, the posted materials are not copyedited and are the sole responsibility of the authors, so questions or comments should be addressed to the corresponding author.
