# Supplementary material for: Direct-Acting Antiviral Therapy and Long-Term Outcomes in Dialysis Patients With Hepatitis C: A Real-world Cohort Study
Source: Open Forum Infect Dis. 2026 Jul 7;13(7):ofag389. doi: 10.1093/ofid/ofag389 (PMC13339124; doi:10.1093/ofid/ofag389)
Supplement: ofag389_Supplementary_Data [file ofag389_supplementary_data.docx]

**Supplementary Table S1.** Diagnostic, procedural, medication, and laboratory codes utilized in the definition of the cohorts.

| Category | Code | Description |
| --- | --- | --- |
| End stage kidney disease |  |  |
| diagnosis | UMLS:ICD10CM:N18.6 | End stage renal disease |
| diagnosis | UMLS:ICD10CM:Z99.2 | Dependence on renal dialysis |
| Hepatitis C virus |  |  |
| laboratory | UMLS:LNC:11011-4 | Hepatitis C virus RNA [Units/volume] (viral load) in Serum or Plasma by NAA with probe detection (at least 0.00 [IU]/mL) |
| laboratory | UMLS:LNC:38180-6 | Hepatitis C virus RNA [log units/volume] (viral load) in Serum or Plasma by NAA with probe detection (at least 1.00 {Log_IU}/mL) |
| laboratory | UMLS:LNC:11259-9 | Hepatitis C virus RNA [Presence] in Serum or Plasma by NAA with probe detection (labResult: Positive) |
| laboratory | UMLS:LNC:20416-4 | Hepatitis C virus RNA [#/volume] (viral load) in Serum or Plasma by NAA with probe detection (at least 0.00 {copies}/mL) |
| laboratory | UMLS:LNC:10676-5 | Hepatitis C virus RNA [Units/volume] (viral load) in Serum or Plasma by Probe with amplification (at least 0.00 [IU]/mL) |
| laboratory | UMLS:LNC:47252-2 | Hepatitis C virus RNA [Log #/volume] (viral load) in Serum or Plasma by NAA with probe detection (at least 1.00 {log_copies}/mL) |
| laboratory | UMLS:LNC:42617-1 | Hepatitis C virus RNA [log units/volume] (viral load) in Serum or Plasma by Probe with signal amplification (at least 1.00 {Log_IU}/mL) |
| laboratory | UMLS:LNC:50023-1 | Hepatitis C virus RNA panel (viral load) in Serum or Plasma by NAA with probe detection (at least 0.00 units) |
| laboratory | UMLS:LNC:49758-6 | Hepatitis C virus RNA [Units/volume] (viral load) in Serum or Plasma by Probe and target amplification method detection limit = 5 iU/mL (at least 0.00 [IU]/mL) |
| laboratory | UMLS:LNC:5010-4 | Hepatitis C virus RNA [Presence] in Blood by NAA with probe detection (labResult: Positive) |
| laboratory | UMLS:LNC:29609-5 | Hepatitis C virus RNA [Units/volume] (viral load) in Serum or Plasma by Probe with signal amplification (at least 0.00 [IU]/mL) |
| Direct-acting antivirals |  |  |
| medication | NLM:RXNORM:85762 | ritonavir |
| medication | NLM:RXNORM:1484911 | sofosbuvir |
| medication | NLM:RXNORM:1799206 | velpatasvir |
| medication | NLM:RXNORM:1591922 | ledipasvir |
| medication | NLM:RXNORM:1940636 | pibrentasvir |
| medication | NLM:RXNORM:1940635 | glecaprevir |
| medication | NLM:RXNORM:1734630 | grazoprevir |
| medication | NLM:RXNORM:1734628 | elbasvir |
| medication | NLM:RXNORM:1597373 | paritaprevir |
| medication | NLM:RXNORM:1597381 | dasabuvir |
| medication | NLM:RXNORM:1597371 | ombitasvir |
| medication | NLM:RXNORM:1939323 | voxilaprevir |
| medication | NLM:RXNORM:1606218 | daclatasvir |
| medication | NLM:RXNORM:1652103 | asunaprevir |
| Kidney transplantation |  |  |
| diagnosis | UMLS:ICD10CM:Z94.0 | Kidney transplant status |
| procedure | UMLS:CPT:1008098 | Renal Transplantation Procedures |
| Dialysis |  |  |
| procedure | UMLS:CPT:90935 | Hemodialysis procedure with single evaluation by a physician or other qualified health care professional |
| procedure | UMLS:CPT:90937 | Hemodialysis procedure requiring repeated evaluation(s) with or without substantial revision of dialysis prescription |
| procedure | UMLS:CPT:90945 | Dialysis procedure other than hemodialysis (eg, peritoneal dialysis, hemofiltration, or other continuous renal replacement therapies), with single evaluation by a physician or other qualified health care professional |
| procedure | UMLS:CPT:90947 | Dialysis procedure other than hemodialysis (eg, peritoneal dialysis, hemofiltration, or other continuous renal replacement therapies) requiring repeated evaluations by a physician or other qualified health care professional, with or without substantial revision of dialysis prescription |
| procedure | UMLS:CPT:90957 | End-stage renal disease (ESRD) related services monthly, for patients 12-19 years of age to include monitoring for the adequacy of nutrition, assessment of growth and development, and counseling of parents; with 4 or more face-to-face visits by a physician or other qualified health care professional per month |
| procedure | UMLS:CPT:90958 | End-stage renal disease (ESRD) related services monthly, for patients 12-19 years of age to include monitoring for the adequacy of nutrition, assessment of growth and development, and counseling of parents; with 2-3 face-to-face visits by a physician or other qualified health care professional per month |
| procedure | UMLS:CPT:90959 | End-stage renal disease (ESRD) related services monthly, for patients 12-19 years of age to include monitoring for the adequacy of nutrition, assessment of growth and development, and counseling of parents; with 1 face-to-face visit by a physician or other qualified health care professional per month |
| procedure | UMLS:CPT:90960 | End-stage renal disease (ESRD) related services monthly, for patients 20 years of age and older; with 4 or more face-to-face visits by a physician or other qualified health care professional per month |
| procedure | UMLS:CPT:90961 | End-stage renal disease (ESRD) related services monthly, for patients 20 years of age and older; with 2-3 face-to-face visits by a physician or other qualified health care professional per month |
| procedure | UMLS:CPT:90962 | End-stage renal disease (ESRD) related services monthly, for patients 20 years of age and older; with 1 face-to-face visit by a physician or other qualified health care professional per month |
| procedure | UMLS:CPT:90965 | End-stage renal disease (ESRD) related services for home dialysis per full month, for patients 12-19 years of age to include monitoring for the adequacy of nutrition, assessment of growth and development, and counseling of parents |
| procedure | UMLS:CPT:90966 | End-stage renal disease (ESRD) related services for home dialysis per full month, for patients 20 years of age and older |
| procedure | UMLS:CPT:90969 | End-stage renal disease (ESRD) related services for dialysis less than a full month of service, per day; for patients 12-19 years of age |
| procedure | UMLS:CPT:90970 | End-stage renal disease (ESRD) related services for dialysis less than a full month of service, per day; for patients 20 years of age and older |
| procedure | UMLS:CPT:90989 | Dialysis training, patient, including helper where applicable, any mode, completed course |
| procedure | UMLS:CPT:90999 | Unlisted dialysis procedure, inpatient or outpatient |
| procedure | UMLS:CPT:1012740 | Dialysis Services and Procedures |
| procedure | UMLS:SNOMED:108241001 | Dialysis procedure |
| procedure | UMLS:CPT:1012752 | Hemodialysis Procedures |
| procedure | UMLS:SNOMED:302497006 | Hemodialysis |
| procedure | UMLS:CPT:1029674 | Dialysis Circuit Procedures |
| diagnosis | UMLS:ICD10CM:Z49.3 | Encounter for adequacy testing for dialysis |
| procedure | UMLS:ICD9CM:39.95 | Hemodialysis |
| procedure | UMLS:ICD10PCS:5A1D60Z | Performance of Urinary Filtration, Multiple (deprecated 2018) |
| procedure | UMLS:ICD10PCS:5A1D00Z | Performance of Urinary Filtration, Single (deprecated 2018) |
| procedure | UMLS:ICD10PCS:3E1M39Z | Irrigation of Peritoneal Cavity using Dialysate, Percutaneous Approach |

**Supplementary Table S2.** Definitions of covariates coding used in this study.

| Code | Description |
| --- | --- |
| AI | Age at Index |
| 2106-3 | White |
| UNK | Unknown Race |
| F | Female |
| 2054-5 | Black or African American |
| M | Male |
| 2131-1 | Other Race |
| 2028-9 | Asian |
| E11.2 | Type 2 diabetes mellitus with kidney complications |
| E11.3 | Type 2 diabetes mellitus with ophthalmic complications |
| E11.4 | Type 2 diabetes mellitus with neurological complications |
| E11.5 | Type 2 diabetes mellitus with circulatory complications |
| E11 | Type 2 diabetes mellitus |
| I10 | Essential (primary) hypertension |
| E78 | Disorders of lipoprotein metabolism and other lipidemias |
| I50 | Heart failure |
| C00-D49 | Neoplasms |
| F17 | Nicotine dependence |
| F10 | Alcohol related disorders |
| E66 | Overweight and obesity |
| K74.6 | Other and unspecified cirrhosis of liver |
| Z94.4 | Liver transplant status |
| C22.0 | Liver cell carcinoma |
| I20-I25 | Ischemic heart diseases |
| B20 | Human immunodeficiency virus [HIV] disease |
| B18.1 | Chronic viral hepatitis B without delta-agent |
| 9045 | Albumin [Mass/volume] in Serum, Plasma or Blood |
| 9014 | Hemoglobin [Mass/volume] in Blood |
| 9037 | Hemoglobin A1c/Hemoglobin.total in Blood |
| 9020 | Platelets [#/volume] in Blood |
| 9044 | Alanine aminotransferase [Enzymatic activity/volume] in Serum, Plasma or Blood |
| 9047 | Aspartate aminotransferase [Enzymatic activity/volume] in Serum or Plasma |
| 9045 | Albumin [Mass/volume] in Serum, Plasma or Blood |
| 9014 | Hemoglobin [Mass/volume] in Blood |
| 9037 | Hemoglobin A1c/Hemoglobin.total in Blood |
| 9020 | Platelets [#/volume] in Blood |

**Supplementary Table S3.** Definitions of outcomes coding used in this study.

| Category | Code | Description |
| --- | --- | --- |
| All-cause mortality |  |  |
| Demographics | Deceased | Deceased |
| Diagnosis | UMLS:ICD10CM:R99 | Ill-defined and unknown cause of mortality |
| Kidney transplantation |  |  |
| Diagnosis | UMLS:ICD10CM:Z94.0 | Kidney transplant status |
| Procedure | UMLS:CPT:1008098 | Renal Transplantation Procedures |
| Liver cancer |  |  |
| Diagnosis | UMLS:ICD10CM:C22.0 | Liver cell carcinoma |
| Cirrhosis |  |  |
| Diagnosis | UMLS:ICD10CM:K74.6 | Other and unspecified cirrhosis of liver |
| Diagnosis | UMLS:ICD10CM:K70.3 | Alcoholic cirrhosis of liver |

**Supplementary Table S4.** Geographic distribution of the study cohort by direct-acting antiviral (DAA) treatment status across the United States, Asia-Pacific, and Europe/Middle East/Africa regions.

|  | Total | DAA (+) | DAA (–) |
| --- | --- | --- | --- |
| United States | 7184 | 1383 | 5801 |
| Asia-Pacific | 429 | 308 | 121 |
| Europe, the Middle East and Africa | 24 | 10 | 14 |

As TriNetX is a continuously updated federated database, cohort counts may vary slightly depending on the timing of data extraction.

**Supplementary Table S5.** Distribution of direct-acting antiviral (DAA) regimens used in the treated cohort.

| DAA drugs | Percentage |
| --- | --- |
| sofosbuvir | 39.21% |
| pibrentasvir | 33.81% |
| glecaprevir | 33.38% |
| velpatasvir | 29.22% |
| grazoprevir | 18.24% |
| elbasvir | 18.11% |
| ledipasvir | 9.06% |
| ritonavir | 8.13% |
| ombitasvir | 2.11% |
| paritaprevir | 2.11% |
| dasabuvir | 1.92% |
| daclatasvir | 1.18% |
| voxilaprevir | 0.81% |
| asunaprevir | 0.62% |

Frequency and distribution of specific direct-acting antiviral (DAA) regimens initiated by patients in the treated cohort. Pangenotypic regimens, which are the current standard of care for patients with end-stage kidney disease regardless of HCV genotype, accounted for the majority of prescriptions.

Note: Percentages sum to >100% because these are often prescribed as combinations (e.g., Glecaprevir and Pibrentasvir are usually the same pill).

**Supplementary Table S6.** Baseline characteristics of patients treated with direct-acting antivirals (DAAs) [DAA (+)] and untreated patients [DAA (–)] in the 6-month landmark analysis cohort before and after propensity score matching.

|  | Before matching | | |  | After matching | | |
| --- | --- | --- | --- | --- | --- | --- | --- |
|  | DAA (+) | DAA (–) | SMD |  | DAA (+) | DAA (–) | SMD |
|  | (n = 1086) | (n = 3378) |  |  | (n = 1024) | (n = 1024) |  |
| **Demographic data** |  |  |  |  |  |  |  |
| Age at index date | 59.3 ± 9.9 | 56.6 ± 11.7 | 0.249 |  | 59.2 ± 9.9 | 59.5 ± 10.8 | 0.030 |
| Male | 748 (68.9%) | 2231 (66.0%) | 0.060 |  | 698 (68.2%) | 714 (69.7%) | 0.034 |
| Race |  |  |  |  |  |  |  |
| Black/African American | 571 (52.6%) | 1960 (58.0%) | 0.110 |  | 550 (53.7%) | 572 (55.9%) | 0.043 |
| White | 288 (26.5%) | 979 (29.0%) | 0.055 |  | 277 (27.1%) | 267 (26.1%) | 0.022 |
| Asian | 106 (9.8%) | 105 (3.1%) | 0.274 |  | 82 (8.0%) | 63 (6.2%) | 0.072 |
| Other race | 36 (3.3%) | 158 (4.7%) | 0.070 |  | 35 (3.4%) | 40 (3.9%) | 0.026 |
| Unknown race | 72 (6.6%) | 141 (4.2%) | 0.109 |  | 67 (6.5%) | 68 (6.6%) | 0.004 |
| Overweight/obesity | 258 (23.8%) | 730 (21.6%) | 0.051 |  | 252 (24.6%) | 246 (24.0%) | 0.014 |
| Smoking | 428 (39.4%) | 1170 (32.8%) | 0.139 |  | 416 (40.6%) | 440 (43.0%) | 0.048 |
| Alcohol use | 224 (20.6%) | 478 (14.2%) | 0.171 |  | 215 (21.0%) | 236 (23.0%) | 0.050 |
| **Comorbidity** |  |  |  |  |  |  |  |
| Type 2 diabetes mellitus | 621 (57.2%) | 1680 (49.7%) | 0.150 |  | 592 (57.8%) | 585 (57.1%) | 0.014 |
| Diabetic kidney complication | 503 (46.3%) | 1321 (39.1%) | 0.146 |  | 479 (46.8%) | 477 (46.6%) | 0.004 |
| Diabetic ophthalmic complication | 158 (14.5%) | 405 (12.0%) | 0.076 |  | 149 (14.6%) | 157 (15.3%) | 0.022 |
| Diabetic neurological complication | 232 (21.4%) | 618 (18.3%) | 0.077 |  | 222 (21.7%) | 221 (21.6%) | 0.002 |
| Diabetic circulatory complication | 90 (8.3%) | 248 (7.3%) | 0.035 |  | 88 (8.6%) | 94 (9.2%) | 0.021 |
| Hypertension | 885 (81.5%) | 2210 (65.4%) | 0.370 |  | 830 (81.1%) | 846 (82.6%) | 0.041 |
| Dyslipidemia | 561 (51.7%) | 1327 (39.3%) | 0.250 |  | 530 (51.8%) | 529 (51.7%) | 0.002 |
| Heart failure | 464 (42.7%) | 1187 (35.1%) | 0.156 |  | 443 (43.3%) | 465 (45.4%) | 0.043 |
| Ischemic heart disease | 477 (43.9%) | 1280 (37.9%) | 0.123 |  | 453 (44.2%) | 468 (45.7%) | 0.029 |
| Neoplasm | 481 (44.3%) | 809 (23.9%) | 0.439 |  | 437 (42.7%) | 430 (42.0%) | 0.014 |
| Hepatocellular carcinoma | 68 (6.3%) | 80 (2.4%) | 0.192 |  | 61 (6.0%) | 56 (5.5%) | 0.021 |
| Cirrhosis | 384 (35.4%) | 579 (17.1%) | 0.423 |  | 347 (33.9%) | 352 (34.4%) | 0.010 |
| Liver transplantation | 57 (5.2%) | 76 (2.3%) | 0.158 |  | 53 (5.2%) | 47 (4.6%) | 0.027 |
| Human immunodeficiency virus | 119 (11.0%) | 100 (3.0%) | 0.318 |  | 99 (9.7%) | 85 (8.3%) | 0.048 |
| Hepatitis B virus | 75 (6.9%) | 62 (1.8%) | 0.250 |  | 57 (5.6%) | 45 (4.4%) | 0.054 |
| **Laboratory data of blood** |  |  |  |  |  |  |  |
| Hemoglobin < 10 g/dL | 771 (71.0%) | 1868 (55.3%) | 0.330 |  | 721 (70.4%) | 736 (71.9%) | 0.032 |
| Hemoglobin levels (g/dL) | 11.0 ± 2.1 | 10.3 ± 2.2 | 0.347 |  | 11.0 ± 2.1 | 10.4 ± 2.2 | 0.294 |
| Albumin < 3.5 g/dL | 821 (75.6%) | 1921 (56.9%) | 0.404 |  | 768 (75.0%) | 788 (77.0%) | 0.046 |
| Albumin levels (g/dL) | 3.6 ± 0.7 | 3.4 ± 0.7 | 0.309 |  | 3.6 ± 0.7 | 3.4 ± 0.7 | 0.295 |
| Platelet ≥ 100 x 10^3^/uL | 1028 (94.7%) | 2463 (72.9%) | 0.617 |  | 966 (94.3%) | 986 (96.3%) | 0.093 |
| Platelet counts (10^3^/uL) | 182.6 ± 81.9 | 196.0 ± 89.7 | 0.157 |  | 184.2 ± 81.9 | 186.2 ± 83.0 | 0.023 |
| Aspartate Aminotransferase ≥ 50 U/L | 609 (56.1%) | 1255 (37.2%) | 0.386 |  | 566 (55.3%) | 582 (56.8%) | 0.031 |
| Aspartate Aminotransferase levels (U/L) | 39.5 ± 67.7 | 60.6 ± 224.7 | 0.127 |  | 39.7 ± 69.6 | 63.0 ± 216.9 | 0.145 |
| Alanine transaminase ≥ 50 U/L | 586 (54.0%) | 1089 (32.2%) | 0.450 |  | 538 (52.5%) | 546 (53.3%) | 0.016 |
| Alanine transaminase levels (U/L) | 35.0 ± 45.4 | 43.5 ± 119.5 | 0.094 |  | 35.1 ± 46.3 | 51.0 ± 155.2 | 0.140 |
| Hemoglobin A1c < 8% | 767 (70.6%) | 1478 (43.8%) | 0.564 |  | 709 (69.2%) | 736 (71.9%) | 0.058 |
| Hemoglobin A1c levels (%) | 6.1 ± 1.6 | 6.4 ± 2.0 | 0.185 |  | 6.1 ± 1.7 | 6.1 ± 1.6 | 0.026 |

SMD = Standardized mean difference

**Supplementary Table S7.** Baseline characteristics of patients treated with direct-acting antivirals (DAAs) [DAA (+)] and untreated patients [DAA (–)] in the 12-month landmark analysis cohort before and after propensity score matching.

|  | Before matching | | |  | After matching | | |
| --- | --- | --- | --- | --- | --- | --- | --- |
|  | DAA (+) | DAA (–) | SMD |  | DAA (+) | DAA (–) | SMD |
|  | (n = 930) | (n = 2872) |  |  | (n = 874) | (n = 874) |  |
| **Demographic data** |  |  |  |  |  |  |  |
| Age at index date | 59.0 ± 9.9 | 56.2 ± 11.8 | 0.256 |  | 59.0 ± 9.9 | 59.1 ± 10.7 | 0.012 |
| Male | 637 (68.5%) | 1882 (65.5%) | 0.063 |  | 595 (68.1%) | 615 (70.4%) | 0.050 |
| Race |  |  |  |  |  |  |  |
| Black/African American | 507 (54.5%) | 1706 (59.4%) | 0.099 |  | 479 (54.8%) | 484 (55.4%) | 0.012 |
| White | 233 (25.1%) | 804 (28.0%) | 0.067 |  | 226 (25.9%) | 235 (26.9%) | 0.023 |
| Asian | 91 (9.8%) | 90 (3.1%) | 0.273 |  | 77 (8.8%) | 54 (6.2%) | 0.100 |
| Other race | 32 (3.4%) | 136 (4.7%) | 0.065 |  | 31 (3.5%) | 39 (4.5%) | 0.047 |
| Unknown race | 57 (6.1%) | 110 (3.8%) | 0.106 |  | 51 (5.8%) | 52 (6.0%) | 0.005 |
| Overweight/obesity | 222 (23.9%) | 647 (22.5%) | 0.032 |  | 213 (24.4%) | 223 (25.5%) | 0.026 |
| Smoking | 375 (40.3%) | 930 (32.4%) | 0.166 |  | 351 (40.2%) | 382 (43.7%) | 0.072 |
| Alcohol use | 184 (19.8%) | 394 (13.7%) | 0.163 |  | 171 (19.6%) | 182 (20.8%) | 0.031 |
| **Comorbidity** |  |  |  |  |  |  |  |
| Type 2 diabetes mellitus | 544 (58.5%) | 1439 (50.1%) | 0.169 |  | 513 (58.7%) | 543 (62.1%) | 0.070 |
| Diabetic kidney complication | 446 (48.0%) | 1134 (39.5%) | 0.171 |  | 420 (48.1%) | 444 (50.8%) | 0.055 |
| Diabetic ophthalmic complication | 147 (15.8%) | 350 (12.2%) | 0.104 |  | 136 (15.6%) | 138 (15.8%) | 0.006 |
| Diabetic neurological complication | 210 (22.6%) | 530 (18.5%) | 0.102 |  | 199 (22.8%) | 212 (24.3%) | 0.035 |
| Diabetic circulatory complication | 75 (8.1%) | 208 (7.2%) | 0.031 |  | 74 (8.5%) | 77 (8.8%) | 0.012 |
| Hypertension | 769 (82.7%) | 1889 (65.8%) | 0.394 |  | 717 (82.0%) | 743 (85.0%) | 0.080 |
| Dyslipidemia | 482 (51.8%) | 1151 (40.1%) | 0.237 |  | 452 (51.7%) | 475 (54.3%) | 0.053 |
| Heart failure | 396 (42.6%) | 1004 (35.0%) | 0.157 |  | 376 (43.0%) | 382 (43.7%) | 0.014 |
| Ischemic heart disease | 403 (43.3%) | 1080 (37.6%) | 0.117 |  | 380 (43.5%) | 395 (45.2%) | 0.035 |
| Neoplasm | 410 (44.1%) | 692 (24.1%) | 0.431 |  | 371 (42.4%) | 380 (43.5%) | 0.021 |
| Hepatocellular carcinoma | 54 (5.8%) | 65 (2.3%) | 0.181 |  | 47 (5.4%) | 47 (5.4%) | < 0.001 |
| Cirrhosis | 322 (34.6%) | 474 (16.5%) | 0.425 |  | 290 (33.2%) | 308 (35.2%) | 0.043 |
| Liver transplantation | 44 (4.7%) | 59 (2.1%) | 0.148 |  | 40 (4.6%) | 38 (4.3%) | 0.011 |
| Human immunodeficiency virus | 108 (11.6%) | 87 (3.0%) | 0.334 |  | 79 (9.0%) | 73 (8.4%) | 0.024 |
| Hepatitis B virus | 66 (7.1%) | 53 (1.8%) | 0.256 |  | 50 (5.7%) | 41 (4.7%) | 0.046 |
| **Laboratory data of blood** |  |  |  |  |  |  |  |
| Hemoglobin < 10 g/dL | 655 (70.4%) | 1581 (55.0%) | 0.322 |  | 613 (70.1%) | 620 (70.9%) | 0.018 |
| Hemoglobin levels (g/dL) | 11.1 ± 2.1 | 10.3 ± 2.2 | 0.360 |  | 11.1 ± 2.1 | 10.4 ± 2.2 | 0.333 |
| Albumin < 3.5 g/dL | 698 (75.1%) | 1616 (56.3%) | 0.404 |  | 651 (74.5%) | 666 (76.2%) | 0.040 |
| Albumin levels (g/dL) | 3.6 ± 0.6 | 3.4 ± 0.7 | 0.319 |  | 3.6 ± 0.6 | 3.4 ± 0.7 | 0.322 |
| Platelet ≥ 100 x 10^3^/uL | 882 (94.8%) | 2083 (72.5%) | 0.633 |  | 827 (94.6%) | 837 (95.8%) | 0.054 |
| Platelet counts (10^3^/uL) | 185.0 ± 81.4 | 197.9 ± 88.4 | 0.151 |  | 186.7 ± 81.7 | 185.8 ± 79.7 | 0.010 |
| Aspartate Aminotransferase ≥ 50 U/L | 519 (55.8%) | 1045 (36.4%) | 0.397 |  | 477 (54.6%) | 505 (57.8%) | 0.065 |
| Aspartate Aminotransferase levels (U/L) | 38.7 ± 68.9 | 57.7 ± 208.1 | 0.123 |  | 38.8 ± 70.9 | 59.9 ± 173.4 | 0.159 |
| Alanine transaminase ≥ 50 U/L | 500 (53.8%) | 922 (32.1%) | 0.448 |  | 455 (52.1%) | 476 (54.5%) | 0.048 |
| Alanine transaminase levels (U/L) | 33.5 ± 35.6 | 41.5 ± 102.9 | 0.104 |  | 33.5 ± 36.3 | 45.0 ± 98.1 | 0.155 |
| Hemoglobin A1c < 8% | 654 (70.3%) | 1263 (44.0%) | 0.552 |  | 602 (68.9%) | 613 (70.1%) | 0.027 |
| Hemoglobin A1c levels (%) | 6.1 ± 1.6 | 6.4 ± 2.0 | 0.157 |  | 6.2 ± 1.6 | 6.2 ± 1.7 | 0.030 |

SMD = Standardized mean difference

**Supplementary Table S8.** Landmark analyses for all-cause mortality to mitigate immortal time bias.

| Analysis Type | HR (95% CI) | P-value |
| --- | --- | --- |
| 6-Month Landmark | 0.81 (0.70–0.94) | < 0.01 |
| 12-Month Landmark | 0.78 (0.66–0.93) | < 0.01 |

This table presents the results of the landmark analyses conducted at 6 and 12 months after the index date.

Notes: Landmark analysis includes only those patients who survived until the specified landmark time point.

Hazard ratios (HR) are estimated using the fully adjusted Model 5.

**Supplementary Table S9.** Sensitivity Analysis Using SVR Proxy and Persistent Viremia.

| Analysis Window | HR (95% CI) | P-value |
| --- | --- | --- |
| SVR Proxy vs. Persistent Viremia |  |  |
| 6-to-12-month window^#^ | 0.57 (0.38–0.85) | < 0.01 |
| Beyond 6 months^#^ | 0.52 (0.37–0.73) | < 0.001 |

This table evaluates the association between documented viral clearance and survival. This analysis minimizes infection misclassification and explores the association of viral eradication with survival.

Notes:

SVR Proxy: Defined as DAA-treated patients with a documented negative HCV RNA test recorded within the 6–12-month window or ≥6 months after treatment initiation.

^#^Persistent Viremia: Defined as untreated patients with at least two positive HCV RNA tests recorded ≥6 months apart, with the second test occurring within the 6–12-month window or ≥6 months after the index date.

**Supplementary Table S10**. Changes in mean albumin and hemoglobin levels from baseline with 95% confidence intervals.

|  | **Albumin** (g/dL) | | | |  | **Hemoglobin** (g/dL) | | | |
| --- | --- | --- | --- | --- | --- | --- | --- | --- | --- |
| **Time** (months) | DAA (+) (N=1458) | DAA (–) (N=1458) | DID | P value |  | DAA (+) (N=1458) | DAA (–) (N=1458) | DID | P value |
| 3 months | –0.01 (–0.06 to 0.04) | –0.11 (–0.17 to –0.05) | 0.10 | 0.01 |  | –0.10 (–0.26 to 0.06) | –0.50 (–0.66 to –0.34) | 0.4 | < 0.001 |
| 6 months | 0.06 (0.01 to 0.11) | –0.05 (–0.11 to 0.01) | 0.11 | < 0.001 |  | 0 (–0.16 to 0.16) | –0.20 (–0.36 to –0.04) | 0.2 | 0.08 |
| 9 months | 0.06 (0.01 to 0.11) | –0.02 (–0.08 to 0.04) | 0.08 | 0.040 |  | 0.10 (–0.06 to 0.26) | –0.20 (–0.36 to –0.04) | 0.3 | 0.01 |
| 12 months | 0.09 (0.04 to 0.14) | –0.01 (–0.07 to 0.05) | 0.10 | 0.01 |  | 0.10 (–0.06 to 0.26) | –0.20 (–0.36 to –0.04) | 0.3 | 0.01 |

Abbreviations: DAA, direct-acting antiviral; DID, difference-in-differences; CI, confidence interval.

**Supplementary Table S11**. Stratified analysis of 5-year all-cause mortality and kidney transplantation by hepatitis B virus (HBV) and Human immunodeficiency virus (HIV) coinfection status in patients treated with direct-acting antivirals (DAA) versus untreated patients.

|  | All-cause mortality | | |  | Kidney transplantation | | |
| --- | --- | --- | --- | --- | --- | --- | --- |
|  | DAA (+) (n/N) | DAA (–) (n/N) | HR (95% CI) |  | DAA (+) (n/N) | DAA (–) (n/N) | HR (95% CI) |
| **HBV status** |  |  |  |  |  |  |  |
| Without HBV | 445/1447 | 599/1447 | 0.59 (0.52-0.67) ^***^ |  | 170/1447 | 107/1447 | 1.39 (1.09-1.77) ^**^ |
| With HBV | 20/57 | 24/57 | 0.65 (0.36-1.19) |  | NA | NA | NA |
| **HIV status** |  |  |  |  |  |  |  |
| Without HIV | 410/1393 | 567/1393 | 0.58 (0.51-0.66) ^***^ |  | 172/1393 | 113/1393 | 1.33 (1.05-1.69) ^*^ |
| With HIV | 43/114 | 48/114 | 0.76 (0.51-1.15) |  | NA | NA | NA |

NA: not applicable. Based on the outcome terms, the patient count is too small, so detailed results cannot be displayed.

N: the number of patients for each stratum; n: the number of patients with outcomes.

HR: Hazard ratio; CI: confidence interval.

^*^: p < 0.05; ^**^: p < 0.01; ^***^: p < 0.001.
